# Making Your Genbank Entry Count

**DOI:** 10.3389/fgene.2012.00123

**Published:** 2012-07-04

**Authors:** David Roy Smith

**Affiliations:** ^1^Department of Botany, Canadian Institute for Advanced Research, University of British ColumbiaVancouver, BC, Canada

The other day I set out on what I thought would be a straightforward task: I wanted to calculate the number of coding nucleotides in each of the plastid genome sequences stored in GenBank. Plastid genomes are found inside the plastid organelles of plants and algae, and most are around 100–200 kb long and contain between 50 and 200 genes. As of May 1, 2012, there are almost 300 complete plastid genome sequences in GenBank. Using the annotations that accompany these sequences, I added up the lengths of all the genes in each genome, not including introns, and divided this number by the total genome length. This should have given me the fraction of coding DNA in the different genomes, but in many cases it did not. A closer look at the sequences revealed the problem: GenBank entries are often poorly annotated.

Indeed, some of the plastid genomes had no annotations for tRNA genes, even though they encoded more than 25 tRNAs. Others contained the right number of genes but lacked annotations for the introns and exons within the genes. There were also examples of open reading frames being annotated as protein-coding regions when they showed no similarity to known proteins. And one entry had no annotations whatsoever – it was just a blank sequence. Certain genomes, however, were exceptionally annotated, containing labels for every exon, intron, functional RNA, pseudogene, and repetitive element as well as for the different nucleotides that undergo post-transcriptional editing. Unfortunately, these types of entries were in the minority.

The low quality of many GenBank entries won't surprise most geneticists, but it should concern them. With recent advancements in nucleotide sequencing technologies, more and more people are depositing molecular sequence data in GenBank, and an even greater number are retrieving these data for use in scientific analyses. Moreover, the amount of data contained in individual entries is on the rise, with whole-genome sequence submissions becoming standard fare. Some scientists, however, are doing a messy job of their GenBank submission because they are in a hurry to get a GenBank accession number so that they can publish their sequence data in an academic journal (proof of GenBank submission is required by most publishers). The irony is that the GenBank entry for a given nucleotide sequence is often more important, from a practical point of view, than the journal article describing that sequence, meaning many researchers are skimming over the most important part of their data presentation. Adding to this problem is that most universities do not teach their science students how to write a GenBank entry.

As an undergraduate in Genetics, I learned how to effectively communicate my results to the scientific community by writing lab reports and giving class presentations, but I was never taught how to impart these results through online sequence repositories. When it came time to prepare my first GenBank entry, as part of my fourth-year honors project, I winged it. Looking back at that submission now, I give it a 4-out-of 10 for quality. I annotated the tRNA- and rRNA-coding regions as “functional RNAs,” but did not label them as “genes,” and I did not even know that pseudogenes should (and could) be included in the entry. A 10-out-of-10 submission would have contained annotations for all of the different genomic architectural features. The National Center for Biotechnology Information does provide some instructions on how to deposit sequences in GenBank, but these are mostly rules for using the submission software rather than guidelines for preparing an effective entry.

During my PhD work, when I regularly devoted whole days to depositing data into GenBank, I asked a distinguished professor of bioinformatics for suggestions on improving my entries. “Tailor your submission to a broad audience,” she said, “especially to people who may not be familiar with your sequence or species. You'll also need to learn the more than 50 different types of annotation that can be added to a GenBank nucleotide sequence, and then decide which of these best fit your data. When possible, always include the strain number as well as the origin and date of isolation of the species from which the sequence was derived.” I agreed with all of her suggestions, and still employ them today. She went on to explain how it can be dangerous to use other entries as a template for annotating your own. “Most of my GenBank mistakes,” she admitted, “are the result of copying the errors of others – this is particularly true for the mislabeling of gene names and symbols. Never assume that what is in GenBank is correct. And remember, a good GenBank entry reflects good work, but a bad one breeds bad science.” Some of my own experiences with using other people's entries for construction organelle genome submissions have resulted in misannotated introns and intronic open reading frames.

A clear and well-annotated GenBank entry is a great way to communicate your results and promote your work. Correctly annotating your sequence will help it show up more often – and at the right times – in databank and BLAST searches. It will also give your entry a greater chance of being downloaded by researchers and employed in meta-analyses. And because GenBank sequences are directly linked to the academic papers that describe them, well-formulated entries can increase the exposure of your published research. All of this can translate into more citations of your scientific papers and help guide future studies – and it will ultimately make life easier for your fellow scientists.

After sifting through the approximately 300 plastid genome sequences in GenBank, I finally picked out the ones with good annotations to use in my analyses on plastid coding DNA. As I did this, I was reminded that to be able to sit at my kitchen table with a laptop computer and explore these genomes, which come from species as diverse as the date palm and the malaria parasite, is a luxury. GenBank is a relatively new invention, and as it (and other major sequence databanks) grows ever larger, I believe that the quality of entries will become increasingly important, as will the ways in which we interact with these data.

